# β-amyloid induces a dying-back process and remote trans-synaptic alterations in a microfluidic-based reconstructed neuronal network

**DOI:** 10.1186/s40478-014-0145-3

**Published:** 2014-09-25

**Authors:** Bérangère Deleglise, Sebastien Magnifico, Eric Duplus, Pauline Vaur, Vanessa Soubeyre, Morgane Belle, Maeva Vignes, Jean-Louis Viovy, Etienne Jacotot, Jean-Michel Peyrin, Bernard Brugg

**Affiliations:** Institut de Biologie Paris Seine (IBPS), 75005 Paris, France; CNRS UMR 8256, Biological Adaptation and Ageing, Team 5: Degenerative Processes in Neurons and Networks, F-75005 Paris, France; CNRS UMR 7102, Neurobiologie des Processus Adaptatifs, F-75005 Paris, France; Sorbonne Universités, UPMC Université Paris 6, F-75005 Paris, France; Institut Curie, Laboratory of Macromolecules and Microsystems in Biology and Medicine, F-75005 Paris, France; CNRS UMR 168, F-75005 Paris, France; Institute of Reproductive and Developmental Biology, Imperial College London, Hammersmith Hospital, London, W12 0NN UK

**Keywords:** Microfluidics, Neuronal network, Axonal degeneration, Tau, Synapse loss

## Abstract

**Introduction:**

Recent histopathological studies have shown that neurodegenerative processes in Alzheimer’s and Parkinson’s Disease develop along neuronal networks and that hallmarks could propagate trans-synaptically through neuronal pathways. The underlying molecular mechanisms are still unknown, and investigations have been impeded by the complexity of brain connectivity and the need for experimental models allowing a fine manipulation of the local microenvironment at the subcellular level.

**Results:**

In this study, we have grown primary cortical mouse neurons in microfluidic (μFD) devices to separate soma from axonal projections in fluidically isolated microenvironments, and applied β-amyloid (Aβ) peptides locally to the different cellular compartments. We observed that Aβ application to the somato-dendritic compartment triggers a “dying-back” process, involving caspase and NAD^+^ signalling pathways, whereas exposure of the axonal/distal compartment to Aβ deposits did not induce axonal degeneration. In contrast, co-treatment with somatic sub-toxic glutamate and axonal Aβ peptide triggered axonal degeneration. To study the consequences of such subcellular/local Aβ stress at the network level we developed new μFD multi-chamber devices containing funnel-shaped micro-channels which force unidirectional axon growth and used them to recreate *in vitro* an oriented cortico-hippocampal pathway. Aβ application to the cortical somato-dendritic chamber leads to a rapid cortical pre-synaptic loss. This happens concomitantly with a post-synaptic hippocampal tau-phosphorylation which could be prevented by the NMDA-receptor antagonist, MK-801, before any sign of axonal and somato-dendritic cortical alteration.

**Conclusion:**

Thanks to μFD-based reconstructed neuronal networks we evaluated the distant effects of local Aβ stress on neuronal subcompartments and networks. Our data indicates that distant neurotransmission modifications actively take part in the early steps of the abnormal mechanisms leading to pathology progression independently of local Aβ production. This offers new tools to decipher mechanisms underlying Braak's staging. Our data suggests that local Aβ can play a role in remote tauopathy by distant disturbance of neurotransmission, providing a putative mechanism underlying the spatiotemporal appearance of pretangles.

**Electronic supplementary material:**

The online version of this article (doi:10.1186/s40478-014-0145-3) contains supplementary material, which is available to authorized users.

## Introduction

The two main hallmarks of Alzheimer’s disease (AD) are extracellular deposits composed of β-amyloid peptide (senile plaques) and intracellular filamentous aggregates composed of self-assembled hyperphosphorylated Tau proteins (neurofibrillary tangles, NFTs). Histopathological studies show that these hallmarks spread, each in their own stereotyped fashion, within specific regions of the brain during disease evolution [1,2]. This progression follows neuro-anatomical pathways and could be the sign of nexopathy-related processes [3]. A large body of evidence indicates that neurons affected in AD follow a dying-back pattern of degeneration, where abnormalities in synaptic function and axonal integrity long precede somatic cell death [4,5]. Since neurons are highly polarized, this raises the question whether local Aβ and Tau protein abnormalities in the vicinity of different neuronal subparts (somatic, dendritic, axonal) lead to local degenerative processes or could initiate distant dysfunction within neurons or even within neuronal networks, through (trans-) synaptic alterations. However, though various mechanisms initiating local primary dysfunctions have been described, the molecular or structural reasons underlying distant breakdown of specific networks and the relationship between amyloid and Tau pathology are still unclear.

Recent advances in the development of microfluidic devices (μFD) facilitate microenvironment development adapted to neuronal structures and subdomains with easy access and control [6,7]. We have previously designed and developed a μFD system to polarize neuronal networks using funnel-shaped micro-channels, also called axonal diodes, allowing the reconstruction of an orientated functional cortico-striatal neuronal network *in vitro* [8]. In the present study, we used a similar μFD-based approach to study the molecular mechanism of neurodegenerative processes in compartmentalized neurons and neuronal networks. By compartmentalizing axon terminals from cell bodies of cortical neurons, we demonstrate that axons are partially resistant to axonal Aβ-peptide exposure, while somatic treatments trigger an anterograde degeneration signal in axons, inducing a *dying-back* pattern. We then reconstructed an oriented cortico-hippocampal network in μFD, and showed that somato-dendritic deposits of Aβ on cortical neurons trigger a rapid cortical presynaptic disconnection concomitant with a glutamate-dependent postsynaptic hippocampal tau-phosphorylation before any axonal and somatic cortical degeneration.

## Results

### Somato-dendritic exposure of cortical neurons to Aβ-peptide induces an axonal dying back pattern

**Figure 1:**
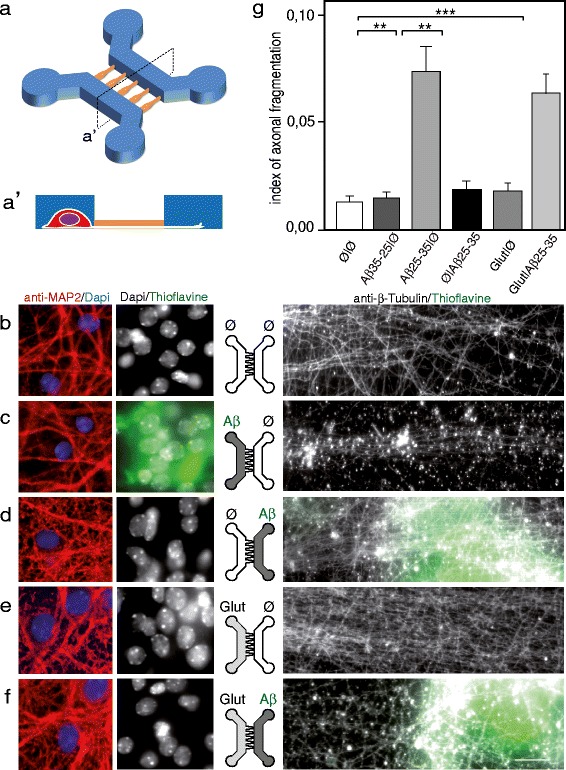
**Somatic applications of Aβ peptides induce a dying-back pattern. a**. Schematic representation of a two-chamber microfluidic device (2-C μFD) with two cell culture chambers interconnected by funnel-shaped micro-channels [8] (**a’**: cross-section of the microfluidic device. A neuron is represented. Note that only axons can reach the right chamber). **b-f**. Fluorescence microscopy analysis of axonal degeneration vs somatic status after addition of Aβ peptide in the left (soma) vs right (distal part of axons) chambers. Cortical neurons were grown for 12 days in two-compartment chips and each chamber was treated for 48 h with Aβ peptides as indicated. The two left micrographs show the somato-dendritic chamber after staining with anti-MAP2 (red), DAPI (blue) and Thioflavine S (green). The right rectangular micrographs show the distal chamber after immunostaining with anti-α-tubulin and labeling with Thioflavine S. Each central scheme represents the device with indicated localization of treatment (left chamber vs right chamber): **b)** control cultures (Ø/Ø), **c)** somato-dendritic treatment with Aβ 25–35 peptide (Aβ/Ø, 30 μM), **d)** axonal treatment with Aβ 25–35 peptide (Ø/Aβ, 30 μM), **e)** somato-dendritic treatment with glutamate (Glut/Ø, 10 μM), **f)** somato-dendritic treatment with glutamate combined with axonal treatment Aβ 25–35 (Glut/Aβ, 10 μM/30 μM). Scale bar: 20 μm. **g)** Quantification of axonal fragmentation. Aβ25-35, glutamate (Glut), and control Aβ35-25 were added, or not (Ø), to the chamber as indicated. Axonal fragmention index was calculated as described in Methods section (n = 3; **P < 0.01; ***P < 0.001, ANOVA).

**Figure 2:**
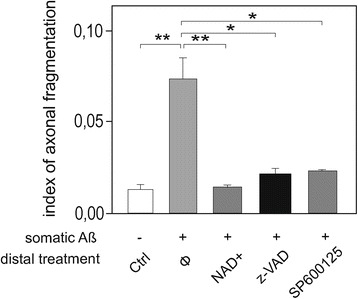
**Axonal administration of NAD+, broad-spectrum caspase inhibitor, and JNK inhibitor reduces** **Aβ-induced axonal degeneration.** Cortical (Cx) neurons were cultured for 12 days in μFD chambers as in Figure 1. Somatic Aβ peptide treatment (48 h) induced a strong axonal fragmentation, which could be reversed by pre-treatment of the axonal compartment with 5 mM NAD^+^, 50 μM z-VAD-fmk or 50 μM SP600125 (n = 5; *P < 0.01; **P < 0.001, ANOVA).

We wondered whether localized β-amyloid deposition on different subcellular compartments (somato-dendritic or axonal) lead to local degenerative signals or to a global neuronal degeneration. Axons were compartmentalized from the somato-dendritic compartment by seeding cortical neurons in μFD devices comprising two chambers connected through asymmetrical micro-channels (2C-μFD diodes). When seeded in the left chamber, cortical neurons projected their axons from the somato-dendritic compartment to the distal compartment through filtering micro-channels (Figure 1a-a’,b). Analysis of dendrites (MAP2, red) and nuclear chromatin visualization (DAPI, blue) indicated that the seeded cortical neurons were healthy after two weeks in culture (Figure 1b, Additional file 1: Figure S1a). Somato-dendritic application of fibrillar Aβ25-35, mimicking amyloid plaques, induced axonal degeneration process (Figure 1c, g). While somato-dentritic application of aggregated Aβ did not affect somatic and dendritic viability (MAP2, red; DAPI, blue; Figure 1c) some local synaptic damage was evidenced in the somatic chamber (MAP2, blue; Vglut1, red; Additional file 1: Figure S1b). Addition of NAD^+^, pharmacological inhibitors of c-Jun N-terminal kinases (JNK, SP600125), or caspases inhibitor (z-VAD-fmk), to the axonal compartment significantly reduced Aβ-induced axonal degeneration (Figure 2). In contrast, distal axonal application of aggregated Aβ failed to induce any significant axonal degeneration in our paradigm (Figure 1d-g). Interestingly, while low dose of somato-dendritic glutamate treatment did not trigger axonal degeneration *per se* (Figure 1e-g, tubulin, grey), the combination of distal Aβ application with this sub-toxic dose of glutamate to the somato-dendritic compartment triggered a massive axonal fragmentation (Figure 1f-g). This suggests that axonal resistance toward local Aβ deposition is tightly controlled by the somato-dendritic behavior and that upstream neurotransmitter dysfunction may precipitate neuronal network collapse.

### Cortical somato-dendritic β-amyloid peptide exposure induces a rapid disconnection of cortico-hippocampal synapses, which precedes axonal degeneration

**Figure 3:**
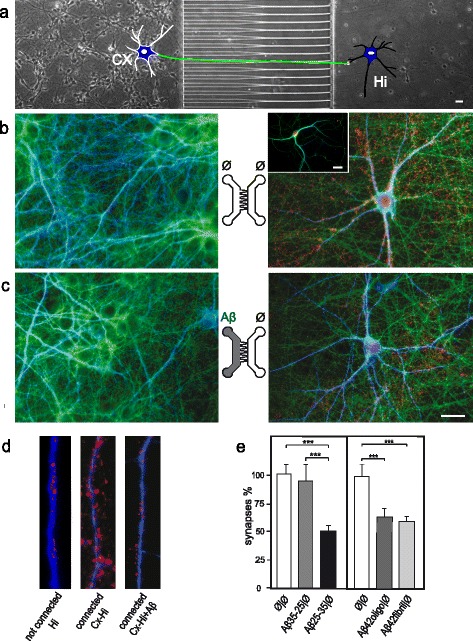
**Cortical Aβ-peptide exposures induce early synaptic loss in a reconstructed cortical-hippocampal network. a**. Phase contrast picture of the microfluidic device comprising funnel-shaped micro-channels allowing unidirectional axonal growth. Cortical neurons (Cx) were seeded in the left chamber, hippocampal neurons (Hi) in the right chamber, and were cultured for 14 days to reconstruct a cortical-hippocampal network. A cartoon representing one cortical neuron connected to one hippocampal neuron is inserted for clarity. **b-e**. Effect of Aβ42 oligomers and Aβ25-35 peptides on synaptic connections. Cortical and hippocampal neurons were cultured in μFD chambers as shown in a. b,c) Representative fluorescence micrographs from somato-dendritic compartment of Cx neurons (left panels) and from Hi neurons in the distal chamber receiving cortical fibers (right panels). Cx chambers were treated with sham (**b**, Ø/ Ø) or Aβ25-35 (**c**, Aβ /Ø). Neurons, axons and synapses were immunodetected using anti-MAP2 (blue), anti-α-tubulin (green), and anti-α-synuclein (red) respectively. Scale bar: 20 μm. Similar presynaptic patterns were observed with anti-synaptophysin labeling (not shown). **d)** Representative higher magnification showing presynaptic clusters (anti-α-synuclein, red) affixed to hippocampal dendrites (anti-MAP2, blue) in hippocampal neurons cultured alone (Hi) connected as in **b** (Hi-Cx) and connected plus treated with Aβ as in **c** (Hi-Cx + Aβ). **e)** Quantification of presynaptic structures affixed to hippocampal dendrites after cortical exposure to oligomeric Aβ1-42 (10 nM), fibrillar Aβ1-42 (10 nM), Aβ25-35 peptides (10 μM), and Aβ35-25 peptides (10 μM). (n = 3; **P < 0.01; ***P < 0.001, ANOVA).

**Figure 4:**
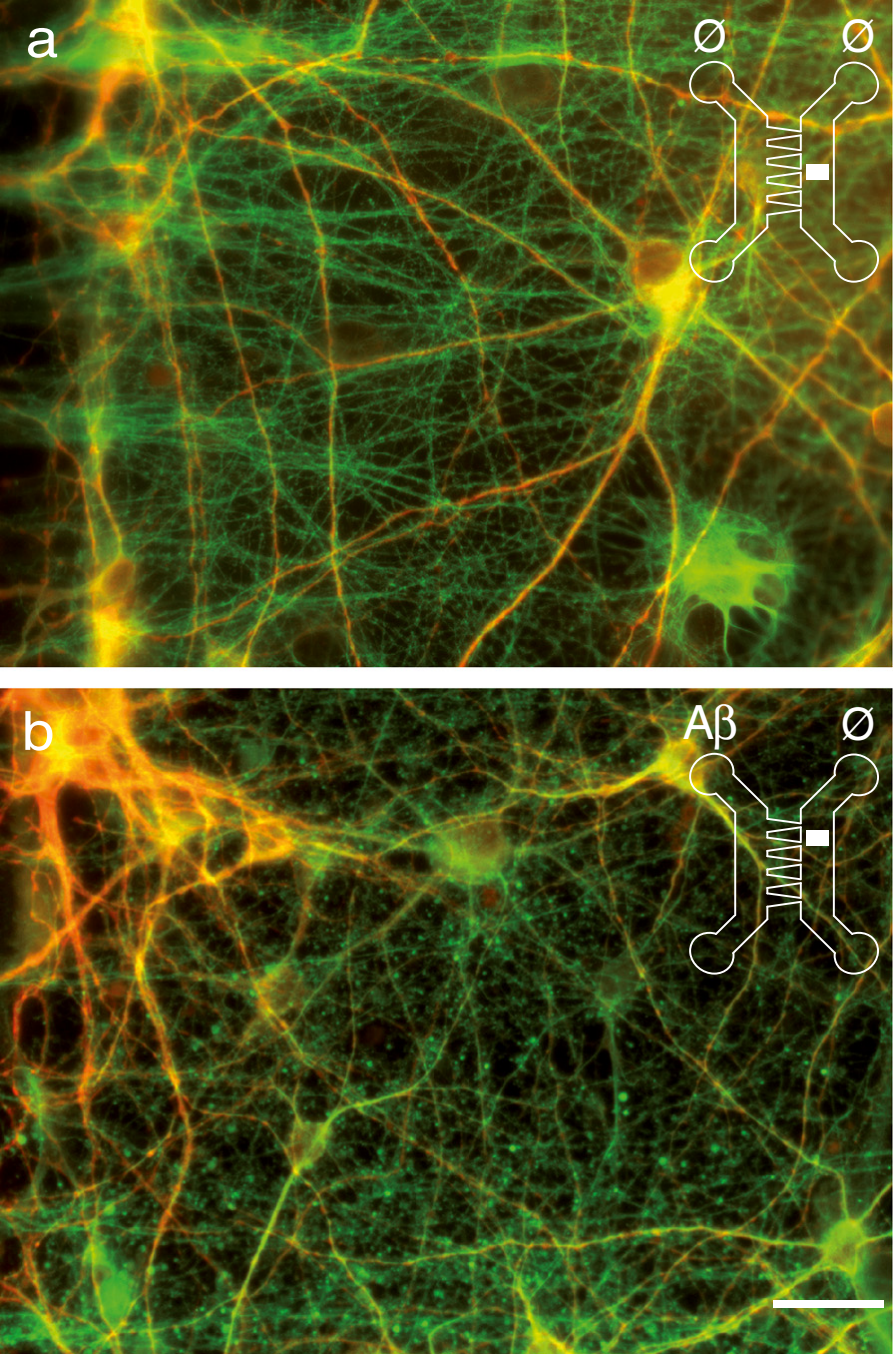
**Exposure of cortical somata to Aβ-peptide induces an axonal loss in a reconstructed cortical-hippocampal network.** Cortical (Cx) and hippocampal (Hi) neurons were cultured for 14 days in μFD chambers as in Figure 2. Hippocampal neurons and projecting cortical axons were immunodetected using anti-MAP2 (red), anti-α-tubulin (green). Cx chambers were treated with sham (**a**, Ø/ Ø) or 10 μM Aβ42 oligomers (**b**, Aβ /Ø) for 48 hours. Representative fluorescence micrographs from the distal hippocampal chamber receiving cortical fibers. Scale bar: 20 μm.

To decipher whether local Aβ might induce remote toxic effect on synapses we used 2C-μFD “axonal diodes” chips that allows reconstructing oriented neuronal networks [8], to create a unidirectional cortico-hippocampal network. When primary cortical neurons (Cx) were cultured at high density in the left chamber of the device and hippocampal neurons (Hi) were seeded at low density in the right chamber (Figure 3a), cortical neurons projected axons (Figure 3b-c; α-tubulin, green) through the funnel-shaped micro-channels and established synapses with hippocampal neurons in the right chamber (Figure 3b-d; MAP2, blue; α-synuclein, red). Thanks to the funnel-shaped μ-channels, hippocampal neurons did not project axons backwards. While hippocampal neurons cultured alone showed few presynaptic cluster along their dendritic shaft (insert in Figure 3b and d), Hippocampal neurons grown in contact with cortical fibers showed high density presynaptic clusters as evidenced by dense α-synuclein (Figure 3d) or VGLUT1 (not shown) staining along the hippocampal dendrites. Application of Aβ25-35 peptide to the Cx compartment induced cortico-hippocampal synapse loss in the Hi compartment within 24 h (Figure 3c-e), whereas cortical axons and soma showed no obvious sign of degeneration (Figure 3c). Similar results were obtained with nanomolar doses of oligomeric or fibrillar Aβ1-42 (Figure 3e) suggesting that this process does not rely on the aggregation state of the peptides. While cortical fibers were still intact 24 h after Aβ (peptide or oligomer) application, at 48 h after treatment, connected axons started to degenerate (Figure 4). We thus observed that the first structural alteration following somato-dendritic Aβ deposits is a distant synapse loss that is followed by delayed axonal degeneration, reminiscent of a dying back process.

### Selective cortical Aβ peptide exposure induces Tau Thr231 phosporylation in a distant fluidically isolated hippocampal neuron through synaptic NMDA receptor-dependent neurotransmission

**Figure 5:**
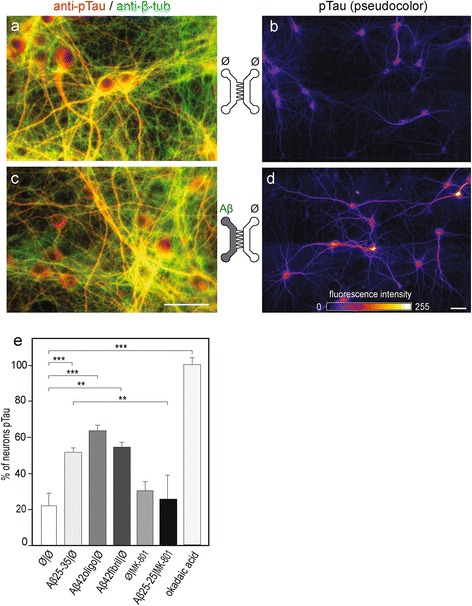
**Cortical Aβ peptide deposition induces glutamate-dependent hippocampal Tau phosphorylation.** Cortical (Cx) and hippocampal (Hi) neurons were cultured in μFD chambers as in Figure 2a. **a**,**c**. Representative fluorescence micrograph ofcortical neurons (left μFD chamber) after immunostaining of phosphorylated Tau (pTau Thr231, red; β-tubulin, green). Effect of Aβ42 oligomers and Aβ25-35 peptides on synaptic connections. Cortical and hippocampal neurons were cultured in μFD chambers as shown in a. b,c) Representative fluorescence micrographs from somato-dendritic compartment of Cx neurons (left panels) and from Hi neurons in the distal chamber receiving cortical fibers (right panels). **b**,**d**. Representative fluorescence micrograph of hippocampal neurons receiving cortical axons after immunostaining of pTau (Thr231; pseudo-colors). **b)** Control conditions (Ø/Ø), **d**) cortical treatment with Aβ25-35 (10 nM) for 24 h. Scale bar: 20 μm. **e**. Quantification of pTau-positive hippocampal neurons after 24 h cortical exposure to fibrillar Aβ25-35, oligomeric Aβ42 or fibrillar Aβ42 (10 nM, each) in the presence or absence of the NMDAR antagonist MK801 (10 μM). The results were compared to pTau-positive neurons induced by exposure of Hi neurons to okadaic acid (1 nM, 24 h) (n = 3 **P < 0.01; ***P < 0.001, ANOVA).

**Figure 6:**
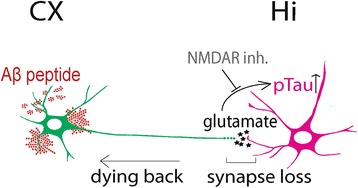
**Hypothetical model of synaptic, post-synaptic and axonal pathological events induced by Aβ in a cortico-hippocampal neuronal network.** β-amyloid application to the somato-dendritic compartment triggers a so-called “dying-back” leading to a rapid cortical presynaptic loss concomitant with a postsynaptic hippocampal Tau-phosphorylation which could be prevented by the NMDA-receptor antagonist, MK-801, before any sign of axonal and somato-dendritic cortical alteration.

It is known that a direct exposure of hippocampal neurons to extracellular soluble Aβ oligomers induce *in vivo* and *in vitro* intracellular Tau hyperphosphorylation (tauHP^+^) and trigger decreased microtubule stability and NFT formation [9]. We consequently examined whether Aβ deposits (oligomeric or fibrillar Aβ1-42, or Aβ25-35) in the Cx chamber could induce distant post-synaptic Tau phosphorylation in the Hi chamber. The antibody recognizing the phospho-threonine 231 epitope detects one of the earliest phosphorylation changes observed in AD patients [10]. After 24 h of Aβ treatment in the Cx chamber we found that phosphorylation levels of Tau Thr231 increased in post-synaptic hippocampal neurons (Figure 5; pTau T231, pseudo-colors). Tau Thr231 phosphorylation was particularly concentrated in the cell bodies and proximal dendrites of hippocampal neurons, rather than in distal dendrites and axons. This effect appeared to be glutamate dependent as phosphorylation was prevented by hippocampal pre-treatment with the NMDA receptor antagonist, MK801 (Figure 5e). Hence, Aβ-mediated disturbance of glutamatergic neurotransmission and concomitant synapse loss can induce Tau phosphorylation in connected hippocampal neurons and subsequently axonal degeneration of cortical fibers, and therefore initiates progressive neuronal network collapse (Figure 6).

## Discussion

A large body of evidence indicates that neurons affected in AD follow a dying-back pattern of degeneration, where such loss of axonal integrity precede somatic cell death and has a profound effect on neuronal network function. However, the molecular mechanism underlying dying-back of neurons and its consequences on the neuronal network in AD remain elusive and difficult to study *in vivo*. Using a new μFD system [8], we modeled for the first time *the* perforant pathway, known to be affected early in AD [1,2]. Somato-dendritic Aβ cortical application within cortico-hippocampal network leads to a rapid presynaptic collapse before cortical axonal or somatic loss [4,5]. Since these synapses were not exposed to Aβ, this suggests that local somato-dendritic Aβ deposits have fast remote toxicity on the unchallenged synapses. This could be due either to a self-propagation [11] and to rapid distribution of Aβ through axonal transport [12] or to the induction of a signal in the soma, which is transmitted through the neuron. We recently described similar distant synapto-toxicity following axotomy [13]. Although no short-term morphological alteration of postsynaptic hippocampal neurons was observed, the Aβ-induced remote loss of cortical presynapses was concomitant to Tau Thr231 phosphorylation in the interconnected postsynaptic hippocampal neurons, and occurred well before axonal and somatic degeneration of cortical neurons. Thus local Aβ deposits generate fast propagation of degenerative signals across networks leading to early dysfunctions in remote areas. Our results show that local somato-dendritic (but not axonal) Aβ triggers distal-to-proximal axonal degeneration before any somato-dendritic abnormalities, a process reminiscent of dying-back pattern observed in various neurodegenerative syndromes [14]. Thus Aβ toxicity depends not only on direct contact but also on the location of its subcellular deposition. Axons are relatively resistant to direct Aβ exposure, which is in line with our recent observation showing that axonal endings are resistant to direct pro-apoptotic insults [6]. Our observation with JNK and caspase pharmacological inhibitors suggest that both enzyme families are implicated locally (within axon) in the process of axonal degeneration, as already observed with other neuronal death inducers [6]. We also shows, for the first time, that axonal addition of NAD^+^ is protective against Aβ-peptide induced axonal degeneration (Figure 2). Since NAD^+^ is a well-established axo-protectant in the context of Wallerian degeneration, this raises the question whether there might be a NAD_+_ controlled molecular pathway implicated in both Aβ-induced axon degeneration and Wallerian degeneration. Therefore NAD^+^ related pathways might offer interesting targets to slow down dying back induced processes.

In some AD patients, increased Aβ is associated with complex disturbances of neuronal activity (e.g. epileptic activity) [15] and neurotransmission dysfunctions have been described in early phases of AD models [16,17]. Such local circuit disturbance might potentially lead to a broader network disruption in remote areas through neuronal projections. Interestingly, we observed that a mild somatic glutamatergic stress exacerbates distant axonal toxicity of Aβ. This suggests that cumulative and multi-focal stresses might play an important role in disease progression, by switching from local (and probably recoverable) minor dysfunctions to extended neuronal alterations like permanent synaptic, axonal or even cell bodies loss. Several non-exclusive mechanisms may account for AD-related spatiotemporal progression in the brain. This may involve neurotrophic factors withdrawal, aberrant neurotransmission, synaptic loss, excitotoxicity and prion-like spread of pathogenic misfolded proteins like trans-synaptic spread of Aβ [3]. Interestingly, in our paradigm, blocking NMDA receptors on hippocampal neurons during cortical somato-dendritic Aβ treatment prevented Tau Thr231 phosphorylation. These results are consistent with studies reporting that Aβ could potentiate potassium-evoked glutamate release from neurons [18,19].

## Conclusion

While brain complexity, with its interconnected neuronal loops, complicates the *in vivo* analysis of pathophysiological initiation and spreading mechanisms, we were able for the first time to evaluate the distant effects of local cortical β-amyloid deposits on neuronal subcompartments and networks in μFD-based reconstructed cortico-hippocampal networks. We show that a strictly local somato-dendritic amyloid trigger is sufficient to recapitulate a dying-back process, and to initiate an oriented neuron-to-neuron progression of pathological events. Aβ peptide accumulation in the somato-dendritic compartment of cortical neurons leads to a fast anterograde propagation of degenerative signals toward endings, resulting in presynaptic collapse. This fast loss of cortical presynapses is associated with early trans-synaptic dysfunction such as NMDAR-dependent tau phosphorylation in postsynaptic hippocampal neurons. Hence, reconstructed cortico-hippocampal μFD networks offer a new tool to decipher mechanisms that could underlie dying back and Braak’s staging.

## Methods

### Primary culture in microfluidic chips

Microfluidic chips were realized as described in [8]. The design used for network reconstruction comprises two culture chambers each connected to two reservoirs and separated by a series of 500 μm-long asymmetrical micro-channels (3 μm high, tapering from 15 μm to 3 μm) [8]. E16 embryos (Swiss mice, Janvier, France) were micro-dissected in GBSS (without CaCl_2_ and MgCl_2_) + 0.1% glucose (Merck), digested with papain (20U/mL) and mechanically dissociated in DMEM containing DNAse. 120.10^3^ cortical cells and 45.10^3^ hippocampal cells were seeded in the chamber on contact with the wide and narrow sides of micro-channels, respectively. Cells were cultured in DMEM glutamax (Invitrogen) supplemented with 10% FBS (PAA), N2, streptomycin/penicillin and B27 (Invitrogen). The culture medium was renewed every 3–5 days. Cortical axon entered the micro-channels and reached the hippocampi-containing chamber in 4–5 days. The cortico-hippocampal oriented network was maintained routinely up to 2–3 weeks *in vitro*.

### Oligomeric and fibrillar Aβ peptide preparations

Oligomeric and fibrillar forms of Aβ1-42 (Tocris Bioscience, MN, USA), were produced according to [20] and controlled by electron microscopy (Additional file 2: Figure S2). Briefly, lyophilized peptides were solubilized at 1 mM in 1, 1, 1, 3, 3, 3,-hexafluoro-2-propanol (HFIP, Sigma Aldrich). After 30 min of incubation at RT, HFIP was evaporated overnight and peptides were dried (Speed Vac, 1 h 4°C). Then, Aβ peptide stock solution were obtained byresolubilizationet 5 mM in dimethylsulfoxide (DMSO, Sigma Aldrich). To obtain oligomers, Aβ stock solution was diluted at 100 μM in phenol-free DMEM-F12 medium (Life technologies), incubated 24 h at 4°C and centrifuged at 20 000 g (10 min; 4°C) before supernatant (soluble Aβ fraction) collection. To obtain fibrils, Aβ stock solution was diluted at 100 μM in HCl 10 mM and then incubated at 4°C for 24 h.

### Electron microscopy

Aβ sample aliquots (10 μM) were allowed to adsorb onto carbon-coated 200-mesh copper grids (EMS, PA, USA) for 2 minutes before blotting off. Then, grids were incubated for 45 seconds in uranyl acetate 2.5% (w/v) to produce a negatively stained protein loaded grid. Images were recorded on a Zeiss 912 omega electron microscope (Carl Zeiss Group, Oberkochen, Germany).

### Treatments

The following compounds were used: Aβ1-42 peptide (1428, Tocris), Aβ25-35 peptide (H-1192, Bachem), control Aβ35-25 peptide (H2964, Bachem), glutamate (G8415, Sigma), MK-801 (0924, Tocris),5 mM NAD^+^ (Sigma), 50 μM z-VAD-fmk (R&D Systems, Minneapolis, MN, USA) and 50 μM SP 600125 (Sigma). Oligomeric and fibrillar forms of Aβ1-42 were produced according to [20] and controlled by electron microscopy. To ensure fluidic isolation between compartments, a hydrostatic pressure difference was generated by over-pressurizing the non-treated chamber as described in [21].

### Immunofluorescence detection

Cultures were fixed (4% PFA, 20 min RT) and immunostained as described in [13]. Primary antibodies included anti-α-tubulin-FITC (F2168, sigma), anti-MAP2 (M4403, Sigma), anti-Synaptophysin (S5768, Sigma), anti-Vglut1, anti-α-synuclein (4179, cell signaling), anti-pTau Thr231 (44746G, Invitrogen). Species-specific secondary antibodies (coupled to Alexa 350, 488, 555) were used (Invitrogen). Images were acquired with an Axio-observer Z1 (Zeiss) fitted with a cooled CCD camera (CoolsnapHQ2, Ropert Scientific) and images were analyzed using ImageJ.

### Quantification of axonal fragmentation

For axonal fragmentation analysis and quantification we used fluorescence microscopy (immuno-detection of tubulin), phase contrast, and picture analysis according to a previously described protocol [22]. Briefly, we used a macro developed in NIH ImageJ software that utilizes the Otsu thresholding algorithm, and a particle analyzer algorithm of ImageJ. The total area of axonal regions with circularity greater than 0.9 was determined and normalized by the total axonal area, which was measured from the thresholded image. This ratio, termed fragmentation index, is an indicator of the average axonal fragmentation level and is used in statistical comparisons. Indices of 0.005, 0.083, and 0.157 correspond to <5%, 50%, and >95% fragmentation, respectively.

### Quantification of synapse loss

Synaptic disconnection was assessed through fluorescence microscopy by counting α-synuclein clusters affixed to MAP2 positive hippocampal dendrites. Images were all obtained with the same acquisition parameters. The images were similarly processed with ImageJ software before being used for quantification: the brightness/contrast of all control images was optimized manually to eliminate the background and to maximize the signal. The means of the minimum and maximum intensities were then calculated in the control condition, after which these settings were applied to all images. The images of the three α-synuclein/α-Tubulin/MAP2 stainings were merged and the resulting image was used to define the zone where hippocampal dendrites were sufficiently innervated by cortical fibres. α-synuclein/MAP2 merges were then used for quantification.

### Quantification of Tau phosphorylation

Tau phosphorylation was assessed by counting the number of neurons presenting pTau levels above a fixed threshold. Images were all obtained using the same acquisition parameters. The images were similarly processed with ImageJ software before being used for quantification: the brightness/contrast of all control images was optimized manually to eliminate the background and to maximize the signal. The means of the minimum and maximum intensities were then calculated in the control condition, after which these settings were applied to all images. All pTau images were then processed with the “Lookup Tables, fire” plugin to visualize the intensity of pTau staining with pseudo-colours. The number of neurons above a fixed colour threshold was then counted and normalized by the total number of neurons to have the percentage of hyperphosphorylated tau neurons.

### Statistical analysis

Differences were assessed by ANOVA, followed, when appropriate, by a post-hoc Bonferoni test. For all analysis * p-value < 0.05; ** p-value < 0.01; *** p–value < 0.001.

## Additional files

Additional file 1: Figure S1. Dendritic morphology of cortical neurons before and after Aβ peptide treatment. Cortical (Cx) neurons were cultured for 14 days in μFD chambers as in Figure 1. Dendrites and pre-synaptic clusters of cortical neurons were immuno-detected using anti-MAP2 (blue), anti-VGLUT1 (red). Cx chambers were treated with sham (**a**, Ø/ Ø) or 10 μM Aβ42 oligomers (**b**, Aβ /Ø) for 48 hours. Representative fluorescence micrographs of the cortical chamber are shown. Scale bar: 20 μm. Additional file 2: Figure S2. Aβ morphology analysed by electron microscopy. Negative stain of TEM images of either Aβ1-42 oligomers **(a)** or fibrils **(b)** and Aβ25-35 aggregates **(c)**.
