# Supplementary figures and images for: β-amyloid induces a dying-back process and remote trans-synaptic alterations in a microfluidic-based reconstructed neuronal network
Source: Acta Neuropathol Commun. 2014 Sep 25;2:145. doi: 10.1186/s40478-014-0145-3 (PMC4193981; doi:10.1186/s40478-014-0145-3)

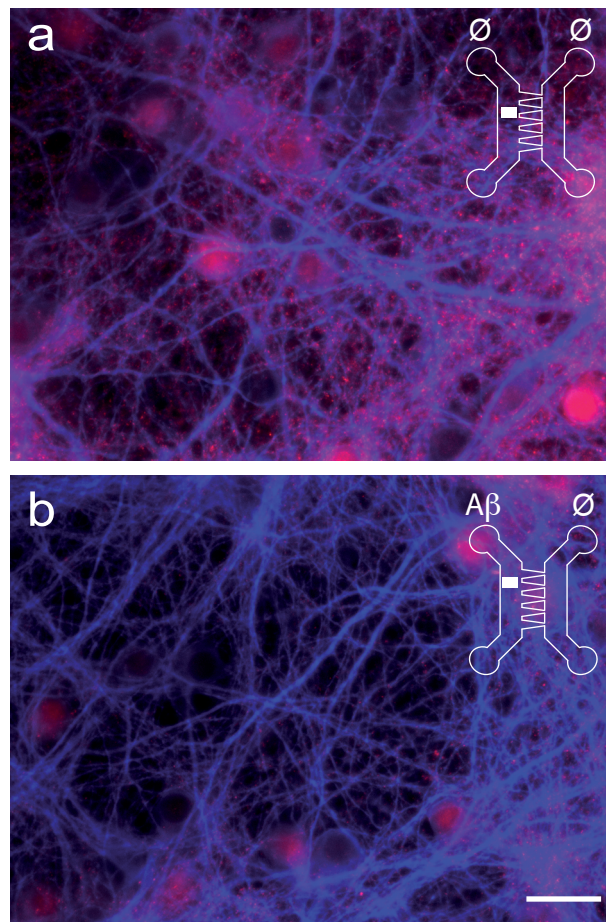

Supp Figure 2, Deleglise et al. 2014

Supplement: Additional file 1: Figure S1. — Dendritic morphology of cortical neurons before and after Aβ peptide treatment. Cortical (Cx) neurons were cultured for 14 days in μFD chambers as in Figure 1. Dendrites and pre-synaptic clusters of cortical neurons were immuno-detected using anti-MAP2 (blue), anti-VGLUT1 (red). Cx chambers were treated with sham (a, Ø/ Ø) or 10 μM Aβ42 oligomers (b, Aβ /Ø) for 48 hours. Representative fluorescence micrographs of the cortical chamber are shown. Scale bar: 20 μm. [file 40478_2014_145_MOESM1_ESM.pdf]

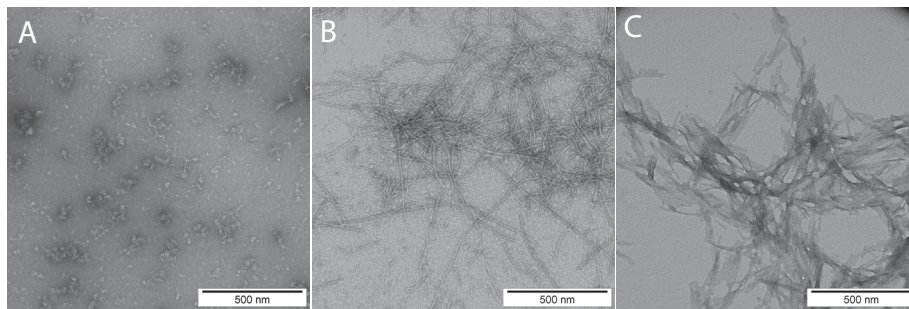

Supp Figure 1, Deleglise et al. 2014

Supplement: Additional file 2: Figure S2. — Aβ morphology analysed by electron microscopy. Negative stain of TEM images of either Aβ1-42 oligomers (a) or fibrils (b) and Aβ25-35 aggregates (c). [file 40478_2014_145_MOESM2_ESM.pdf]
